# Mediterranean diet as a strategy for preserving kidney function in patients with coronary heart disease with type 2 diabetes and obesity: a secondary analysis of CORDIOPREV randomized controlled trial

**DOI:** 10.1038/s41387-024-00285-3

**Published:** 2024-05-16

**Authors:** Alicia Podadera-Herreros, Antonio P. Arenas-de Larriva, Francisco M. Gutierrez-Mariscal, Juan F. Alcala-Diaz, Ana Ojeda-Rodriguez, Fernando Rodriguez-Cantalejo, Magdalena P. Cardelo, Diego Rodriguez-Cano, Jose D. Torres-Peña, Raul M. Luque, Jose M. Ordovas, Pablo Perez-Martinez, Javier Delgado-Lista, Jose Lopez-Miranda, Elena M. Yubero-Serrano

**Affiliations:** 1 Unidad de Gestión Clinica Medicina Interna, Lipids and Atherosclerosis Unit, Maimonides Institute for Biomedical Research in Córdoba, Reina Sofia University Hospital, 14004 University of Córdoba, Córdoba, Spain; 2 CIBER Physiopathology of Obesity and Nutrition (CIBEROBN), Institute of Health Carlos III, Madrid, Spain; 3 Biochemical Laboratory, Reina Sofia University Hospital, 14004 Córdoba, Spain; 4 Department of Cell Biology, Physiology and Immunology, University of Córdoba, Maimonides Institute for Biomedical Research in Córdoba, Reina Sofia University Hospital, University of Córdoba, 14004 Córdoba, Spain; 5 Nutrition and Genomics Laboratory, JM-USDA Human Nutrition Research Center on Aging at Tufts University, Boston, MA 02111 USA; 6 Precision Nutrition and Obesity Program, IMDEA Alimentación, 28049 Madrid, Spain

**Keywords:** Kidney diseases, Translational research

## Abstract

**Background:**

Type 2 diabetes mellitus (T2DM) is recognized an independent risk factor for chronic kidney disease (CKD). The precise contribution and differential response to treatment strategies to reduce kidney dysfunction, depending on whether obesity is present alongside T2DM or not, remain to be fully clarified. Our objective was to improve our understanding of how obesity contributes to kidney function in patients with T2DM and coronary heart disease (CHD), who are highly predisposed to CKD, to assign the most effective dietary approach to preserve kidney function.

**Methods:**

1002 patients with CHD and estimated glomerular filtration rate (eGFR)≥30 ml/min/1.73m^2^, were randomized to consume a Mediterranean diet (35% fat, 22% MUFA, < 50% carbohydrates) or a low-fat diet (28% fat, 12% MUFA, > 55% carbohydrates). Patients were classified into four groups according to the presence of T2DM and/or obesity at baseline: Non-Obesity/Non-T2DM, Obesity/Non-T2DM, Non-Obesity/T2DM and Obesity/T2DM. We evaluated kidney function using serum creatinine-based estimated glomerular filtration rate (eGFR) and urinary albumin-to-creatinine ratio (uACR) before and after 5-years of dietary intervention.

**Results:**

Patients with Obesity/T2DM had the lowest baseline eGFR and the highest baseline uACR compared to non-diabetics (*p* < 0.05). After dietary intervention, the Mediterranean diet induced a lower eGFR decline in patients with Obesity/T2DM, compared to a low-fat diet but not in the other groups (*p* = 0.014). The Mediterranean diet, but not the low-fat diet, also reduced uACR only in patients with Obesity/T2DM (*p* = 0.024).

**Conclusions:**

Obesity provided an additive effect to T2DM resulting in a more pronounced decline in kidney function compared to T2DM alone when compared to non-diabetics. In patients with concomitant presence of T2DM and obesity, with more metabolic complications, consumption of a Mediterranean diet seemed more beneficial than a low-fat diet in terms of preserving kidney function. These findings provide valuable insights for tailoring personalized lifestyle modifications in secondary prevention of cardiovascular disease.

**Trial registration:**

URL, http://www.cordioprev.es/index.php/en. Clinicaltrials.gov number, NCT00924937

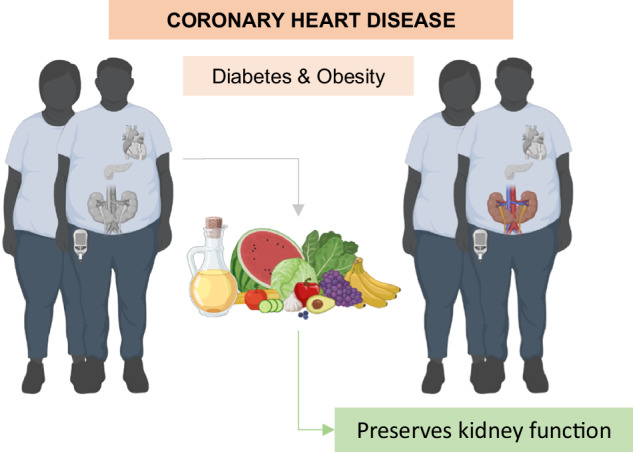

## Background

The prevalence of cardiometabolic conditions has risen due to different factors such as longer life expectancy, lifestyle modifications and improved diagnostic tools [1–3]. In fact, chronic kidney disease (CKD), which affects over 10% of the total adult population, is marked by a gradual decline of kidney function, leading to end-stage renal disease, and imposing significant costs on the healthcare system [4]. Type 2 diabetes (T2DM) is recognized as an independent risk factor for CKD, where persistent or uncontrolled hyperglycemia triggers various pathways contributing to kidney dysfunction, including excessive reactive oxygen species production, hypoxia, mitochondrial dysfunction and inflammation [5, 6]. However, it is not only T2DM but also obesity that contributes to kidney impairment [7], either indirectly through systemic arterial hypertension or directly through lipid accumulation in the glomerulus leading to structural changes such as increased permeability, glomerulosclerosis or albuminuria [8, 9]. Furthermore, obesity has been associated with reduced estimated glomerular filtration rate (eGFR), a comprehensive indicator of kidney function [10, 11]. Nonetheless, the impact of obesity whether accompanied by diabetes or not, on kidney dysfunction remains uncertain [12, 13]. As a result, this unclear relationship may lead to ineffective treatment approaches depending on whether obesity and T2DM coexist or are present separately.

Lifestyle modifications, particularly dietary changes, are proposed as primary strategies in the management of T2DM and obesity [14]. In this regard, different results from prospective observational studies and clinical trials have underscored the significance of individual nutrients, specific foods and overall dietary patterns in preventing T2DM and its associated comorbidities. It emphasizes the importance of the quality of dietary fats and carbohydrates consumed, rather than solely focusing on their quantity [15]. Diets characterized by a high intake of vegetables, fruit, whole grains, nuts, legumes, and low in red/processed meats, refined grains, and sugar-sweetened beverages have demonstrated efficacy in the reduction of the risk of developing T2DM and the improvement of glycemic control [16–19]. We have recently found that the long-term consumption of a healthy dietary pattern (a Mediterranean diet or a low-fat diet) reduced the risk of T2DM development in patients with coronary heart disease (CHD) [20]. Interestingly, in the context of this study, consumption of these diets also increased the probability of diabetes remission in patients with newly-diagnosed T2DM [21]. Furthermore, the Mediterranean diet, but not the low-fat diet, was associated with a slower decline in kidney function, as measured by eGFR, particularly in patients with T2DM [22].

Taking all the above factors into in account, this study aimed to more precisely assess the impact of obesity, in combination with T2DM, to kidney impairment in patients with CHD who are highly predisposed to CKD, in order to assign the most suitable dietary strategy (a Mediterranean diet and/or a low-fat diet) and thus further preserve kidney function.

## Methods

### Design and study population

This work was performed within the framework of the CORDIOPREV study (Clinicaltrials.gov number NCT00924937), a single center, prospective, randomized, single-blind and controlled dietary intervention clinical trial that includes 1002 patients with CHD. This study was developed at Reina Sofia University Hospital in Córdoba, Spain. The volunteers followed one of two different dietary models, a Mediterranean or a low-fat diet, for 7 years, in addition to their conventional treatment for CHD. Details of the rationale, study methods, inclusion and exclusion criteria, as well as a description of the cardiovascular risk factors and the patients’ baseline characteristics have been previously described [23]. Patients provided written informed consent to participate in the study. The trial protocol was approved by the ethics committee of Reina Sofia University Hospital in Cordoba (No. 1496/27/03/2009), following the principles of the Helsinki Declaration and good clinical practices.

In the CORDIOPREV study, the assessment of kidney function and the analysis of the impact of T2DM and obesity on kidney impairment were not defined before the start of the study.

### Randomization and dietary intervention

Randomization was performed by the Andalusian School of Public Health. More details on the randomization procedures were previously described [23]. In addition to the conventional treatment for CHD, each patient was randomly assigned to follow one of these dietary patterns: (a) the Mediterranean diet, with a minimum of 35% of total calories from fat [22% monounsaturated fat (MUFA), 6% polyunsaturated fat (PUFA), and < 10% saturated fat (SFA)], 15% proteins, and a maximum of 50% carbohydrates and (b) a low-fat, high complex carbohydrate diet, as recommended by the National Cholesterol Education Program, with < 30% of total calories from fat (12–14% MUFAs, 6–8% PUFAs, < 10% SFAs), ≥ 55% from carbohydrates and 15% from protein. In both diets, the cholesterol content was adjusted to < 300 mg/day. While both study diets incorporated foods from all major food groups, no specific total calorie restriction was set. Comprehensive details on dietary assessment and follow-up visits have been published previously [23, 24]. The research included no interventions to increase physical activity or weight loss. Participants in both intervention groups were given the same intensive dietary counselling. The follow-up period of the study was 5 years. Detailed information on the specific recommended diets as well as mean baseline and changes in energy and nutrient intake after the 5-years intervention period for both dietary patterns has been described elsewhere [24].

In this study, although both dietary patterns share common characteristics in key components (e.g., abundant intake of fruit, vegetables, whole grains and legumes), an increase in the consumption of oily fish, nuts, and extra virgin olive oil was found, along with a reduction in the consumption of harmful foods such as red/processed meats and pastries/commercial bakery products in patients adhering to the Mediterranean diet compare to the low-fat diet. Adherence to the Mediterranean diet was determined by the 14-item MEditerranean Diet Adherence Screener (MEDAS) while that of the low-fat diet was evaluated by a 9-item dietary screener. Details of the dietary adherence assessment have been previously published [24].

### Anthropometric measurements and laboratory tests

At 8.00 am, following a 12 h fast, the patients were admitted to the laboratory for anthropometric and biochemical tests [weight, body mass index (BMI), systolic blood pressure (SBP), diastolic blood pressure (DBP), HDL-cholesterol, LDL-cholesterol, total cholesterol, triglycerides, high sensitive C-reactive protein (hsCRP), fasting glucose and insulin, homeostasis model assessment of insulin resistance (HOMA-IR) and hemoglobin A1c (HbA1c) as described previously [25]. Serum and urine creatinine were determined by the modified Jaffé colorimetric method [26] and measured by spectrophotometry (BioSystems SA, Barcelona, Spain).

### Evaluation of kidney function

Kidney function was evaluated by measuring serum creatinine (sCr)-based eGFR, calculated using the CKD-Epi (CKD Epidemiology Collaboration) equation as previously published [27, 28].

All 1002 patients completed the evaluation of kidney function at baseline (all of them with eGFR ≥ 30 ml/min/1.73 m^2^, as one of the inclusion criteria of the study [23]). Out of these 1002 patients, 69 did not complete the 5-year follow-up due to withdrawal or death. From these 933 patients, 74 patients had no eGFR data available and 6 patients showed extreme values of total energy intake: < 500 kcal/day or > 3500 kcal/day for women and < 800 kcal/day or > 4000 kcal/day for men, following the criteria proposed by Willet [29]. In this context, the kidney function of 853 patients was evaluated after the 5-year follow-up period. The screening and randomization flow-chart of the CORDIOPREV study and the evaluation of kidney function are shown in Figure S1. Baseline characteristics of those patients who completed the evaluation of kidney function study (during follow-up) compared to patients who did not complete it have been published previously [22].

We also evaluated the ratio of urinary albumin to creatinine (uACR), both at baseline and after the 5-year follow-up period. This ratio was computed by dividing the concentration of urinary albumin concentration by that of creatinine, in mg/g. Using this method, which is based on a spot urine test, produces results which are comparable to those obtained using a 24 h urine collection [30]. Patients who had their kidney function evaluated at 5 years (*n* = 853, with available eGFR data) also had uACR measurements available both at baseline and after 5 years of dietary intervention.

### Criteria for Type 2 diabetes mellitus and obesity status

T2DM was defined according to the American Diabetes Association (ADA) diagnosis criteria [31]. Obesity was defined as a BMI ≥ 30 kg/m^2^. In order to evaluate the specific contribution of obesity, along with T2DM or not, on eGFR, we classified the population into four different groups: Non-Obesity/Non-T2DM, Obesity/Non-T2DM, Non-Obesity/T2DM and Obesity/T2DM.

### Statistical analyses

The data are presented, for the continuous variables, as the mean ± standard error of the mean (SE) and, for the categorical variables, as proportions. The biochemical variables were assessed for normal distribution and variables that presented skewed distribution were normalized by log10 transformation. We compared the categorical variables using Chi-Square tests, and assessed between-group changes with an unpaired t-test or univariate ANOVA for the continuous variables, as required. Significant correlations were studied using bivariate Pearson correlation methods. Test of linear trend across the groups were performed assigning the median value of eGFR and uACR to each group and treating the variable as continuous. To evaluate the variation in data according to diet and time (from baseline to 5 years), we used repeated-measures ANOVA analyses, with a Bonferroni post-hoc test. We compared Δchanges (between 5-year and baseline values) in eGFR between groups with univariate ANOVA adjusted by required cofounders. We conducted a generalized linear model (GLM) using ΔeGFR, as dependent variable, to evaluate potential interaction effects of diet on changes in eGFR according to the presence of diabetes and/or obesity. The effect size was based on GLM coefficients (β) with a 95% confidence interval. The statistical analyses were carried out using SPSS version 22.0 for Windows (SPSS Inc., Chicago, IL, USA).

## Results

### Baseline anthropometric and biochemical characteristics of the study population

The baseline characteristics of patients with CHD whose kidney function was assessed at baseline and who were assigned to randomized dietary groups, have been published previously [22]. We observed that patients who did not undergo the follow-up evaluation of kidney function study were older, with a greater prevalence of current smoking compared to those who completed the assessment [22].

Baseline characteristics of the patients according to presence or absence of obesity are shown in Table S1. Patients with obesity exhibited higher uACR, HbA1c, HOMA-IR and fasting glucose, insulin and hsCRP levels and lower HDL-cholesterol levels compared to patients without obesity (all *p* < 0.001). No differences in baseline eGFR were observed between patients with and without obesity. Moreover, patients with obesity showed a higher percentage of presence of T2DM and, consequently, higher antidiabetic treatment, compared to patients without T2DM (all *p* < 0.001).

The baseline characteristics according to the presence or absence of T2DM have been published previously [22]. Briefly, patients with T2DM were older, and contained a higher percentage of patients with obesity and under treatment for diabetes and/or hypertension, compared to patients without T2DM (all *p* < 0.05). Patients with T2DM also had lower eGFR and higher uACR and HOMA-IR, fasting glucose, insulin, and HbA1c and triglyceride levels in comparison to patients without T2DM (all *p* < 0.05).

**Fig. 1:**
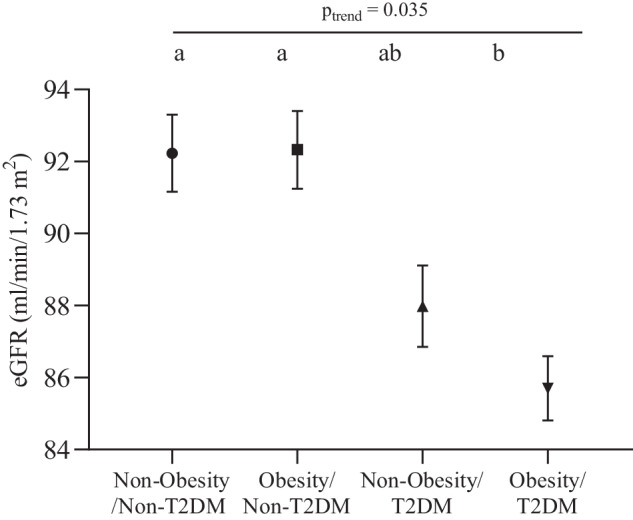
Effect of the presence of T2DM and/or obesity at baseline on eGFR in patients with CHD. Data are presented as mean ± standard error of the mean. Variables were compared using the analysis of variance (univariate ANOVA) adjusted by hypertension, smoking and drinking habits and use of lipid-lowering drugs. Differences were significant when *p* < 0.05. Different common letter denote significant differences between groups (*p* < 0.05). Test of linear trends across the groups were performed assigning the median value of eGFR and uACR to each group and treating the variable as continuous (p_trend_). Non-obesity/Non-T2DM (*n* = 233), Obesity/Non-T2DM (*n* = 229), Non-obesity/T2DM (*n* = 208) and Obesity/T2DM (*n* = 332). CHD coronary heart disease; T2DM, type 2 diabetes mellitus; eGFR, estimated glomerular filtration rate; uACR, urine albumin-creatinine ratio.

When T2DM and obesity were considered together, these patients (Obesity/T2DM) exhibited the highest HbA1c, HOMA-IR and fasting glucose, insulin, hsCRP and triglyceride levels (all *p* < 0.001) (Table 1). Regarding the parameters related to kidney function, patients with Obesity/T2DM showed lower eGFR compared to their counterparts without T2DM (*p* < 0.001) with a significant trend across the groups (*p* = 0.035) (Fig. 1). Moreover, patients with Obesity/T2DM exhibited the highest uACR (129.00 ± 21.57 mg/g) compared to the other groups (Non-Obesity/Non-T2DM = 15.85 ± 25.55 mg/g; Obesity/Non-T2DM = 21.82 ± 25.83 mg/g, Non-Obesity/T2DM = 58.08 ± 27.04 mg/g; all *p* < 0.001) with a significant trend across the groups (*p* < 0.001, Figure S2).

**Table 1:** Baseline clinical and metabolic characteristics of the total patients with CHD according to the presence or absence of obesity and/or T2DM.

	Non-obesity/Non-T2DM (*n* = 233)	Obesity/Non-T2DM (*n* = 229)	Non-obesity/T2DM (*n* = 208)	Obesity/ T2DM (*n* = 332)	*p*-value
Age, years	57.2^a^ (0.6)	58.1^a^ (0.6)	61.3^b^ (0.6)	61.1^b^ (0.5)	<0.001
Male, %	83.6	85.1	84.5	79.2	0.223
Weight, kg	75.0^a^ (0.6)	91.5^b^ (0.9)	75.8^a^ (0.6)	93.6^b^ (0.7)	<0.001
BMI, kg/m^2^	27.0^a^ (0.1)	33.6^b^ (0.2)	27.6^a^ (0.1)	34.5^c^ (0.2)	<0.001
Hypertension, %^1^	65.7	65.2	68.4	73.3	0.133
Fasting glucose, mg/dL	91.6^a^ (0.7)	95.2^a^ (0.6)	124.2^b^ (2.6)	135.6^c^ (2.7)	<0.001
HbA1c, %	5.86^a^ (0.02)	5.93^a^ (0.02)	7.11^b^ (0.08)	7.4^c^ (0.1)	<0.001
HOMA-IR	1.59^a^ (0.07)	2.47^a^ (0.11)	3.0^a^ (0.2)	6.39^b^ (0.77)	<0.001
Fasting insulin, mU/L	7.16^a^ (0.31)	10.5^b^ (0.5)	9.6^b^ (0.6)	14.9^c^ (0.9)	<0.001
Total cholesterol, mg/dL)	161.91 (2.21)	161.2 (2.0)	156.5 (2.3)	157.0 (1.6)	0.124
LDL-cholesterol, mg/dL	92.3 (1.7)	90.9 (1.7)	85.9 (1.9)	86.0 (1.4)	0.109
HDL-cholesterol, mg/dL	45.7^a^ (0.7)	43.1^b^ (0.6)	41.4^c^ (0.7)	39.8^c^ (0.5)	<0.001
Triglycerides, mg/dL	113.6^a^ (3.7)	128.0^b^ (4.2)	131.8^b^ (4.8)	148.9^c^ (3.6)	<0.001
Smoking habits, %					
Never smokers	27.2^ab^	19.3^a^	26.7^ab^	30.2^b^	0.037
Current smokers	9.1	7.9	14.6	9.1	0.090
Former smokers	63.8^ab^	72.8^a^	58.7^b^	60.7^b^	<0.001
Medication use, %					
Lipid-lowering drugs	90.7	92.0	92.6	91.3	0.902
Oral antidiabetic drugs	0.0^a^	0.0^a^	57.8^b^	68.9^b^	<0.001
Antihypertensive drugs	85.3^a^	87.7^ab^	91.3^ab^	94.0^b^	<0.001

Data are mean (standard error) or percentage of participants. *P*-value for comparisons between groups calculated with Chi-square tests for categorical variables or One-way ANOVA test for quantitative variables.

Obesity was defined as a BMI ≥ 30 kg/m^2^.

^1^Hypertension was defined as a systolic blood pressure ≥ 140 mm Hg, a diastolic blood pressure ≥ 90 mm Hg, or the use of antihypertensive therapy.

T2DM was defined as being diagnosed as diabetic before the start of the study and those diagnosed by a fasting blood glucose level ≥ 126 mg/dL on two occasions, or a 2 h plasma glucose level ≥ 200 mg/dL during a 75 g oral glucose-tolerance test, during the first procedures of the study.

Different common letter superscripts denote significant differences between groups (*p* < 0.05).

*CHD* Coronary heart disease, *T2DM* type 2 diabetes mellitus, *BMI* Body max index, *HbA1c* Glycated hemoglobin, *HOMA-IR* Homeostatic Model Assessment for Insulin Resistance, *LDL-cholesterol* low density lipoprotein-cholesterol, *HDL-cholesterol* Low density lipoprotein-cholesterol.

### Associations between parameters of kidney function and those related to glucose metabolism and obesity

Correlations between kidney function parameters and T2DM and obesity-related parameters at baseline in the total study population are shown in Table S2. We found that baseline eGFR was inversely associated with HbA1c, fasting glucose (all *p* < 0.001) and insulin levels (*p* = 0.058) but not with BMI. Moreover, uACR was directly associated with HbA1c, fasting glucose and insulin levels and BMI (all *p* < 0.001).

### Dietary effect on kidney function in presence or absence of T2DM and/or obesity

**Fig. 2:**
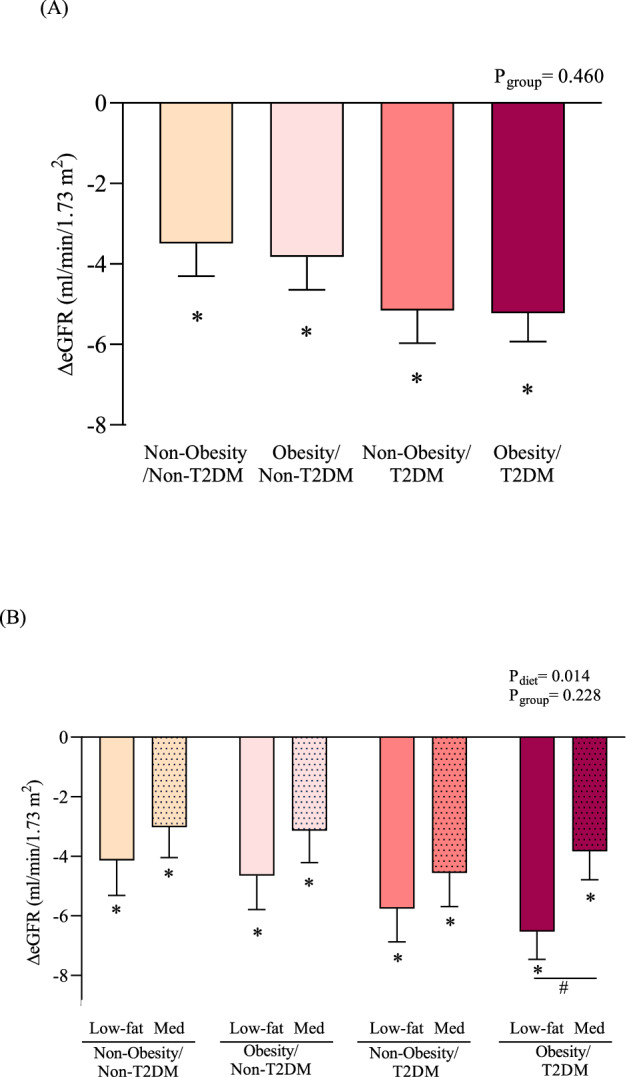
Effect of dietary intervention on eGFR in patients with CHD according to the presence or absence of obesity and/or T2DM. **A** After both dietary patterns and (**B**) After each dietary pattern independently. Non-obesity/Non-T2DM (*n* = 203), Obesity/Non-T2DM (*n* = 197), Non-Obesity/T2DM (*n* = 178) and Obesity/T2DM (*n* = 275). Data are presented as Δ changes in eGFR (mL/min/1.73 m^2^) produced between post- and preintervention ± standard error of the mean. Variables were compared using the analysis of variance (univariate ANOVA) adjusted by age, sex, baseline hypertension, baseline eGFR categories, anti-diabetic drugs and changes in energy intake. Differences were significant when *p* < 0.05. * Significant differences between post and pre-intervention.^#^Significant differences between Mediterranean diet and low-fat diet. Global p-values: p (group): presence in obesity and/or T2DM group effect and p (diet): diet effect. eGFR, estimated glomerular filtration rate; CHD, coronary heart disease; T2DM, type 2 diabetes mellitus.

The effect of dietary intervention (Δ changes produced between post- and pre-intervention) on eGFR according to the presence or absence of T2DM and/or obesity is shown in Fig. 2.

In all the groups of patients, eGFR declined after dietary intervention period, regardless of the type of diet (Mediterranean diet or low-fat diet), compared to baseline (all *p* < 0.001) without differences among groups (Fig. 2A). However, when the type of dietary model was considered, consuming the Mediterranean diet led to a lower reduction in eGFR in comparison to the low-fat diet only in patients with Obesity/T2DM (*p* = 0.032). In fact, after consuming the Mediterranean diet, the decline rate of eGFR was −2.698 ml/min/1.73 m^2^ lower when compared with the low-fat diet, in this type of patients, during the 5-year follow-up period (*p* = 0.032) (Fig. 2B).

To evaluate potential interaction effects of diet on changes in eGFR according to the presence of diabetes and/or obesity, we conducted a GLM (Table 2). In patients without T2DM, no significant interaction was found between the diet and the presence of obesity. However, in patients with T2DM, we found a significant interaction between diet and presence of obesity. Patients with obesity consuming the low-fat diet show a β = −3.009, *p* = 0.033 for changes in eGFR compared to patients with obesity consumed the Mediterranean diet (as reference).

**Table 2:** General linear model for interaction analysis in eGFR after dietary intervention in patients with CHD.

	Patients without T2DM			Patients with T2DM			
	Beta	SE	*p*		Beta	SE	*p*
Intercept	−3.486	0.928	0.000^1^		−3.797	1.014	0.000^1^
Low-fat diet x Non-Obesity	−0.265	1.395	0.850		−2.083	1.602	0.194
Low-fat diet x Obesity	−0.548	1.369	0.689		−3.009	1.412	0.033^1^
Mediterranean diet x Non -Obesity	1.038	1.280	0.418		−1.544	1.602	0.335
Mediterranean diet x Obesity	REF	REF	REF		REF	REF	REF

Interaction effect tested by generalized linear model (GLM) using ΔeGFR as dependent variable.

^1^Differences were considered to be significant when *p* < 0.05. *REF* Reference group.

*CHD* Coronary heart disease, *T2DM* type 2 diabetes mellitus, *eGFR* estimated glomerular filtration rate, *SE* standard error.

Regarding the analysis of another parameter related to kidney function, uACR, according to the presence/absence of T2DM and obesity, after dietary intervention, we found that the Mediterranean diet reduced uACR (∆uACR −1.62 ± 12.98 mg/g), compared to the low-fat diet (∆uACR 18.51 ± 12.65 mg/g; *p* = 0.024) in patients with Obesity/T2DM only, but not in the other groups of patients. Moreover, in patients with Obesity/T2DM, the Mediterranean diet was able to maintain fasting glucose levels after 5-years of follow-up, while the low-fat diet increased it. No significant differences were found in the other clinical parameters, according to each dietary pattern, in this group of patients (Table 3). We also found no significant differences in antidiabetic and antihypertensive treatments between baseline and after 5 years of follow-up in each study group of patients and considering each dietary pattern separately (Table S3).

**Table 3:** Mean baseline values and changes in clinical variables after 5 years of dietary intervention in patients with CHD.

	Non-Obesity/Non-T2DM		Obesity/Non-T2DM		Non-Obesity/T2DM		Obesity/ T2DM	
	Low-fat diet	Mediterranean diet	Low-fat diet	Mediterranean diet	Low-fat diet	Mediterranean diet	Low-fat diet	Mediterranean diet
BMI,kg/m^2^	−0.20 ± 0.29	0.25 ± 0.24	−0.64 ± 0.27^1^	0.39 ± 0.25^2^	−0.34 ± 0.28	0.02 ± 0.28	−1.07 ± 0.22^1^	−0.81 ± 0.22^1^
LDL-c, mg/dL	−3.78 ± 3.07	−2.05 ± 2.59	−6.95 ± 2.98^1^	−3.00 ± 2.72	−3.88 ± 3.11	−2.10 ± 3.01	−2.69 ± 2.42	−3.86 ± 2.46
HDL-c, mg/dL	−0.29 ± 0.98	−1.67 ± 0.83^1^	−1.30 ± 0.95	−0.08 ± 0.87	−2.25 ± 0.99^1^	−0.22 ± 0.95	−2.08 ± 0.77^1^	−2.05 ± 0.78^1^
Triglycerides, mg/dL	−10.2 ± 7.72	−11.1 ± 6.51^1^	0.13 ± 7.49	−2.61 ± 6.82	−14.4 ± 7.83	−7.13 ± 7.63	−6.05 ± 6.10	−4.70 ± 6.20
Fasting glucose, mg/dL	2.28 ± 3.85	2.28 ± 3.23	0.64 ± 3.73	4.48 ± 3.41	5.18 ± 3.87	6.77 ± 3.76	13.7 ± 3.05^1^	2.94 ± 3.10^2^
Fasting insulin, mU/L	1.64 ± 3.0	0.98 ± 2.53	1.05 ± 2.92	3.29 ± 2.64^1^	9.81 ± 3.03^1^	3.65 ± 2.98^1^	7.00 ± 2.42^1^	5.76 ± 2.43^1^
HbA1c, %	−0.23 ± 0.10^1^	−0.26 ± 0.09^1^	−0.32 ± 0.10^1^	−0.16 ± 0.09	−0.33 ± 0.10^1^	−0.41 ± 0.10^1^	−0.21 ± 0.08^1^	−0.29 ± 0.08^1^
SBP, mm Hg	−0.09 ± 2.70	−1.35 ± 2.27	−2.15 ± 2.60	8.26 ± 2.38^1,2^	1.92 ± 2.62	1.24 ± 2.62	−2.94 ± 2.07	1.36 ± 2.12
DBP, mm Hg	−3.09 ± 1.28^1^	−2.82 ± 1.07^1^	−2.69 ± 1.23^1^	1.06 ± 1.12^2^	−3.02 ± 1.24^1^	−2.41 ± 1.24	−2.92 ± 0.98^1^	−1.90 ± 1.00
hsCRP, mg/L	−0.14 ± 0.58	0.49 ± 0.46	0.29 ± 0.52^1^	0.61 ± 0.49	0.32 ± 0.57	1.77 ± 0.53	0.23 ± 0.44	0.48 ± 0.44

Data are presented as mean and Δ-change produced between post- and preintervention ± standard error of the mean. Data are mean (standard error) or percentage of participants.

Differences were significant when *p* < 0.05.

^1^Significant differences between post and pre-intervention.

^2^Significant differences between low-fat and Mediterranean diet.

*CHD* Coronary heart disease, *BMI* Body max index, *LDL-c* low density lipoprotein-cholesterol, *HDL-c* high density lipoprotein-cholesterol, *HbA1c* glycated hemoglobin, *SBP* Systolic blood pressure, *DBP* Diastolic blood pressure, *hsCRP* high-sensitivity C-reactive protein.

With the aim to determine whether these findings were related to the effect of weight loss, after dietary intervention, we evaluated changes in weight in these patients. However, no significant difference was found in weight loss between the two diets in patients with Obesity/T2DM (Mediterranean diet: ∆weight = −2.83 ± 0.60; low-fat diet: ∆weight = −2.18 ± 0.61 kg, *p* = 0.450).

Moreover, we wondered if the effect of diet observed in changes in eGFR in patients with Obesity/T2DM could be due to differences in dietary adherence among the groups of patients. For this reason, we evaluated differences in dietary adherence according to the presence of T2DM and/or obesity. Mean baseline values and changes in adherence after 5 years of dietary intervention are shown in Table S4. All the groups of patients showed significant increases in the dietary adherence after 5 years, compared to baseline (all *p* < 0.05). No differences were found either in baseline values or ∆changes in dietary adherence among the groups of patients.

## Discussion

To the best of our knowledge, no previous studies have evaluated the impact of obesity on T2DM and its effect on kidney function in patients with CHD, despite the fact that these patients are at an increased risk for kidney complications. In this randomized and controlled dietary clinical trial, we found that the presence of obesity had an additive effect on T2DM, resulting in a greater impairment of kidney function with lower eGFR compared to their counterparts without T2DM and the highest uACR. Long-term Mediterranean diet consumption, in comparison to a low-fat diet, produced a lower deterioration of kidney function (lower eGFR decline and uACR) only in patients with more metabolic complications (e.g., with concomitant T2DM and obesity). In addition to age, the presence of certain chronic diseases determines a progressive decline in eGFR [32]. In fact, several studies have argued that kidney function is impaired by diabetes, pointing to an average annual decline in eGFR of ≈2 mL/min/1.73 m^2^ [33]. However, while there is an independent association between obesity and reduced eGFR in individuals with and without CKD [34], other studies suggest that metabolic imbalance contributes more to kidney function impairment than elevated body fat alone [35, 36].

A recent observational study pointed out the presence of metabolic syndrome, rather than obesity measures (BMI or waist circumference), as being associated with an accelerated eGFR decline in the general population [13]. These findings are in line with our results, where patients with T2DM (in the presence of obesity) showed reduced eGFR compared to patients without T2DM patients (with and without obesity). Moreover, eGFR was inversely correlated with HbA1c and fasting glucose levels but not with BMI, suggesting a greater contribution of metabolic imbalance or a loss of metabolic flexibility to kidney dysfunction than the influence of body mass.

The close interconnection between T2DM and obesity can make it difficult to determine the precise impact of each disease on the impairment of kidney function, and as a result, it may be challenging to develop effective therapies and treatments. Over the last few years, lifestyle modifications, particularly dietary habits, have been recognized as the first-line strategies in the management of T2DM and its complications [37, 38]. In fact, in the context of the CORDIOPREV study, we have recently found that the long-term consumption of a healthy dietary pattern (a Mediterranean or a low-fat diet) reduced the risk of developing T2DM in patients with CHD [20, 39]. Moreover, we have also demonstrated that the Mediterranean diet, compared to a low-fat diet, produced a positive effect on delaying the decline of eGFR in patients with CHD. However, when the presence or absence of T2DM was considered, this effect was observed only in those patients with T2DM [22]. In this study, the effect of the Mediterranean diet in slowing down the decline of eGFR was more pronounced when T2DM and obesity coexisted rather than when these pathologies were considered independently, with no differences in weight loss between the diets. Furthermore, no changes in dietary adherence to either diet were detected among the groups, regardless of the presence of diabetes and/or obesity.

While the precise molecular mechanisms remain unclear, we can hypothesize that the impact made by the Mediterranean diet on eGFR-based kidney function in our population could be attributed to its established modulation of cardiometabolic risk factors, such as a better control of plasma lipid levels, reduction of hypertension and attenuation of oxidative stress and inflammation [40–42]. Current data suggest that vascular changes associated with endothelial dysfunction play an important role in the progression of renal impairment [43]. In line with this, our recent findings indicate that the Mediterranean diet is capable of restoring endothelial dysfunction through a more evenly balanced endothelial homeostasis [44, 45]. The Mediterranean diet, characterized by an abundance of minimally processed natural foods, together with extra virgin olive oil (EVOO) as the primary source of fat, provides MUFA and other minor components with antioxidant and anti-inflammatory properties. This dietary pattern has been associated with reduced insulin resistance and has improved endothelium-dependent vasodilatation in patients with T2DM [46]. Another possible mechanism by which the Mediterranean diet affects kidney function could be by reducing the circulating levels of advanced glycation end products (AGEs). In fact, our recent studies have demonstrated that consumption of a Mediterranean dietary pattern can lead to reduced circulating AGEs levels in patients with CHD and T2DM, as well as in patients with metabolic syndrome and in elderly adults [25, 47, 48]. These cytotoxic compounds, known for their oxidant and pro-inflammatory properties, play an important role in the pathogenesis of renal disorders, particularly when the patient suffers from T2DM and obesity [49, 50].

Our study has several key strengths. First, it employs a randomized design that encompasses two different dietary patterns with a large sample of patients. Moreover, it is an in-depth dietary intervention study which compares two healthy dietary patterns with an excellent dietary adherence [24, 51]. However, one potential limitation could be the absence of a standard control diet. Nevertheless, as our study was carried out within the framework of the CORDIOPREV trial, which involves patients with diagnosed CHD in the secondary prevention stage, it was not deemed ethically viable to use a control diet during the design phase of the study. After studying a range of guidelines and international consensus documents [52–55], we opted for testing the long-term effect of two active intervention groups (a low-fat diet *vs* a Mediterranean diet), both of which had proven benefits in patients with high cardiovascular risk. Nevertheless, the study also has certain limitations. This research is based on a long-term, well-controlled dietary intervention, ensuring the quality of the study, but it may not reflect the level of compliance in a free-living population. Furthermore, the findings are confined to patients with T2DM and CHD, which limits their general applicability to other populations. Additionally, it is important to note that the evaluation of kidney function was not the primary endpoint of the CORDIOPREV study, which means that it was impossible to link causality from our findings. Finally, eGFR was not determined through direct measurement such as inulin or iothalamate, or 24-h urinary creatinine clearance, as these costly and time-consuming procedures are not suited to the routine detection of kidney disease.

In summary, our data provide evidence for the greater contribution of obesity and T2DM combined than of T2DM alone to the impairment of kidney function in patients with CHD compared to patients without T2DM. Our study also supports the notion that the response to two healthy dietary interventions on eGFR differs depending on whether obesity and T2DM coexist or are present separately. We propose that patients with both T2DM and obesity, who also suffer more metabolic complications, may respond better in terms of preserving kidney function to the beneficial effects of the consumption of the Mediterranean diet, compared to a low-fat diet. These findings highlight the potential of dietary strategies as clinical and therapeutic tools, leading to a better understanding of personalized health, particularly, in the context of secondary cardiovascular disease prevention.

### Supplementary information

Screening and randomization flow-chart of the CORDIOPREV study and the evaluation of kidney function

Effect of the presence of diabetes and/or obesity at baseline on uACR in patients with coronary heart disease.

Baseline characteristics of the population according to obesity status

Correlations between kidney function parameters and T2DM and obesity related parameters at baseline in the total study population.

Antidiabetic and antihypertensive treatment regimens at baseline and after 5-years of follow-up

Mean baseline values and changes in adherence after 5 years of dietary intervention.

## Data Availability

Collaborations with the CORDIOPREV study are open to Biomedical Institutions, always following an accepted proposal for scientific study. Depending on the nature of the collaboration, electronic data, hard copy data, or biological samples should be provided. All collaborations will be made after a collaboration agreement. Terms of the collaboration agreement will be specific for each collaboration, and the extent of the shared documentation (i.e., anonymized participant data, data dictionary, biological samples, hard copy, or other specified data sets) will also be specifically agreed in the light of each work.
