# Correction: Mediterranean diet as a strategy for preserving kidney function in patients with coronary heart disease with type 2 diabetes and obesity: a secondary analysis of CORDIOPREV randomized controlled trial

**DOI:** 10.1038/s41387-024-00304-3

**Published:** 2024-06-12

**Authors:** Alicia Podadera-Herreros, Antonio P. Arenas-de Larriva, Francisco M. Gutierrez-Mariscal, Juan F. Alcala-Diaz, Ana Ojeda-Rodriguez, Fernando Rodriguez-Cantalejo, Magdalena P. Cardelo, Diego Rodriguez-Cano, Jose D. Torres-Peña, Raul M. Luque, Jose M. Ordovas, Pablo Perez-Martinez, Javier Delgado-Lista, Jose Lopez-Miranda, Elena M. Yubero-Serrano

**Affiliations:** 1 Unidad de Gestión Clinica Medicina Interna, Lipids and Atherosclerosis Unit, Maimonides Institute for Biomedical Research in Córdoba, Reina Sofia University Hospital, 14004 University of Córdoba, Córdoba, Spain; 2 CIBER Physiopathology of Obesity and Nutrition (CIBEROBN), Institute of Health Carlos III, Madrid, Spain; 3 Biochemical Laboratory, Reina Sofia University Hospital, 14004 Córdoba, Spain; 4 Department of Cell Biology, Physiology and Immunology, University of Córdoba, Maimonides Institute for Biomedical Research in Córdoba, Reina Sofia University Hospital, University of Córdoba, 14004 Córdoba, Spain; 5 Nutrition and Genomics Laboratory, JM-USDA Human Nutrition Research Center on Aging at Tufts University, Boston, MA 02111 USA; 6 Precision Nutrition and Obesity Program, IMDEA Alimentación, 28049 Madrid, Spain

**Keywords:** Kidney diseases, Translational research

Correction to: *Nutrition and Diabetes* 10.1038/s41387-024-00285-3; published online 16 May 2024

In this article the statement in the Funding information section was incorrectly given as ‘“......PI21/00383 to Elena M Yubero-Serrano), integrated into the framework of the National Plan for Scientific Research, Technological Development and Innovation 2013-2016, co-financed by the Instituto de Salud Carlos III (ISCIII) of Spain and also by the General Directorate for Assessment and Promotion of Research and the EU’s EuropIean Regional Development Fund (FEDER)

‘and should have read

“......PI21/00383 to Elena M Yubero-Serrano), funded by Instituto de Salud Carlos III (ISCIII) and co-funded by the European Union”

The original article has been corrected.

