# Supplementary material for: Mediterranean diet as a strategy for preserving kidney function in patients with coronary heart disease with type 2 diabetes and obesity: a secondary analysis of CORDIOPREV randomized controlled trial
Source: Nutr Diabetes. 2024 May 16;14:27. doi: 10.1038/s41387-024-00285-3 (PMC11099022; doi:10.1038/s41387-024-00285-3)
Supplement: Supplementary file 1 — Screening and randomization flow-chart of the CORDIOPREV study and the evaluation of kidney function [file 41387_2024_285_MOESM1_ESM.docx]

**Figure S1.** Screening and randomization flow-chart of the CORDIOPREV study and the evaluation of kidney function. CORDIOPREV, CORonary Diet Intervention with Olive oil and cardiovascular PREVention.
