# Supplementary material for: Mediterranean diet as a strategy for preserving kidney function in patients with coronary heart disease with type 2 diabetes and obesity: a secondary analysis of CORDIOPREV randomized controlled trial
Source: Nutr Diabetes. 2024 May 16;14:27. doi: 10.1038/s41387-024-00285-3 (PMC11099022; doi:10.1038/s41387-024-00285-3)
Supplement: Supplementary file 2 — Effect of the presence of diabetes and/or obesity at baseline on uACR in patients with coronary heart disease. [file 41387_2024_285_MOESM2_ESM.docx]

**Figure S2.** Effect of the presence of diabetes and/or obesity at baseline on uACR in patients with coronary heart disease.


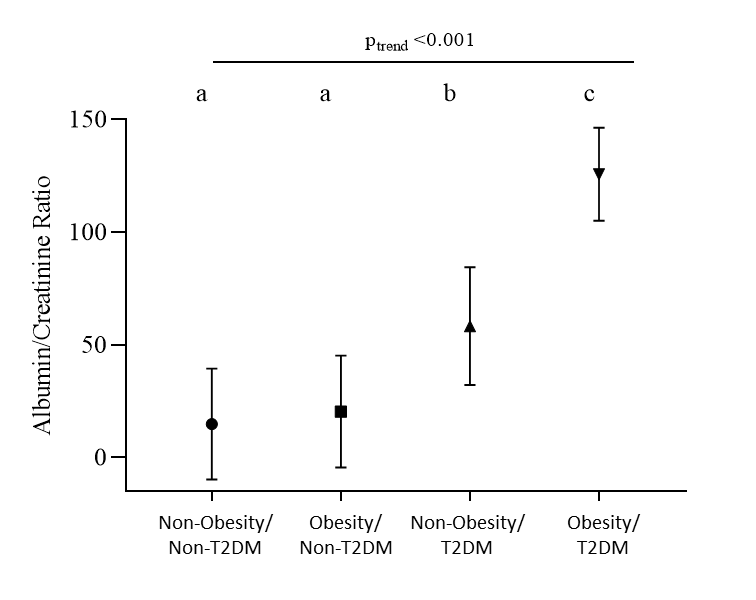


Data are presented as mean ± standard error of the mean. Variables were compared using the analysis of variance (univariate ANOVA) adjusted by hypertension, smoking and drinking habits and use of lipid-lowering drugs. Non-Obesity/Non-T2DM (n=233), Obesity/Non-T2DM (n = 229), Non-Obesity/T2DM (n = 208) and Obesity/T2DM (n = 332). Differences were considered to be significant when p < 0.05. ^abc^ Different common letter superscripts denote significant differences (p < 0.05). Significance for uACR levels were tested on log10 transformed due to skewed distribution Abbreviation: uACR, urine albumin-creatinine ratio.
