# Supplementary material for: Mediterranean diet as a strategy for preserving kidney function in patients with coronary heart disease with type 2 diabetes and obesity: a secondary analysis of CORDIOPREV randomized controlled trial
Source: Nutr Diabetes. 2024 May 16;14:27. doi: 10.1038/s41387-024-00285-3 (PMC11099022; doi:10.1038/s41387-024-00285-3)
Supplement: Supplementary file 3 — Baseline characteristics of the population according to obesity status [file 41387_2024_285_MOESM3_ESM.docx]

**Table S1.** Baseline characteristics of the population according to obesity status*^1^*

|  | **Non-Obesity**  **(n = 441)** | | | **Obesity**  **(n = 561)** | ***p* value *** |
| --- | --- | --- | --- | --- | --- |
| Age, years | | 59.2 (0.4) | | 59.9 (0.4) | 0.817 |
| Male, % | | 84.0 | | 81.6 | 0.314 |
| Weight, kg | | 75.4 (0.4) | | 92.7 (0.6) | <0.001 |
| BMI, kg/m^2^ | | 27.3 (0.1) | | 34.1 (0.2) | <0.001 |
| Hypertension, %^§^ | | 67.0 | | 70.0 | 0.304 |
| eGFR, ml/min/1.73 m^2^ | | 90.2 (0.8) | | 88.4 (0.7) | 0.359 |
| uACR, mg/g | | 35.3 (6.6) | | 82.8 (20.7) | <0.001 |
| Fasting glucose, mg/dL | | 107.0 (1.5) | | 119.1 (1.8) | <0.001 |
| HbA1c, % | | 6.45 (0.05) | | 6.81 (0.05) | <0.001 |
| HOMA-IR | | 2.23 (0.11) | | 4.76 (0.46) | <0.001 |
| Fasting insulin, mU/L | | 8.32 (0.33) | | 13.1 (0.6) | <0.001 |
| T2DM, %^†^ | | 47.0 | | 59.2 | <0.001 |
| Total cholesterol, mg/dL | | | 159.4 (1.6) | 158.7 (1.2) | 0.756 |
| LDL-cholesterol, mg/dL | | 89.3 (1.3) | | 88.0 (1.1) | 0.452 |
| HDL-cholesterol, mg/dL | | 43.7 (0.5) | | 41.1 (0.4) | <0.001 |
| Triglycerides, mg/dL | | 122.1 (3.0) | | 140.4 (2.8) | 0.100 |
| ***Smoking habits, %*** | |  | |  |  |
| Never smokers | | 26.9 | | 25.8 | 0.717 |
| Current smokers | | 11.6 | | 8.6 | 0.111 |
| Former smokers | | 61.4 | | 65.7 | 0.184 |
| ***Medication use, %*** | |  | |  |  |
| Lipid-lowering drugs | | 91.6 | | 91.6 | 1.000 |
| Oral antidiabetic drugs | | 27.2 | | 40.8 | <0.001 |
| Antihypertensive drugs | | 88.1 | | 91.4 | 0.090 |

Data are mean (standard error) or percentage of participants. P value for comparisons between groups calculated with Chi-square tests for categorical variables or independent t-test test for quantitative variables. *Non-obese group vs. obese group, *p* < 0.05.

*^1^* Obesity was defined as a BMI ≥30 kg/m^2^ ^§^ Hypertension was defined as a systolic blood pressure ≥ 140 mm Hg, a diastolic blood pressure ≥ 90 mm Hg, or the use of antihypertensive therapy.

^†^T2DM was defined as being diagnosed as diabetic before the start of the study and those diagnosed by a fasting blood glucose level ≥ 126 mg/dL on two occasions, or a 2-h plasma glucose level ≥ 200 mg/dL during a 75-g oral glucose-tolerance test, during the first procedures of the study.

BMI, body max index; eGFR, estimated glomerular filtration rate; uACR, urinary albumin creatinine ratio; HbA1c, glycated hemoglobin; HOMA-IR, Homeostatic Model Assessment for Insulin Resistance; T2DM, type 2 diabetes mellitus; LDL-cholesterol, low density lipoprotein-cholesterol; HDL-cholesterol, low density lipoprotein-cholesterol.
