# Supplementary material for: Mediterranean diet as a strategy for preserving kidney function in patients with coronary heart disease with type 2 diabetes and obesity: a secondary analysis of CORDIOPREV randomized controlled trial
Source: Nutr Diabetes. 2024 May 16;14:27. doi: 10.1038/s41387-024-00285-3 (PMC11099022; doi:10.1038/s41387-024-00285-3)
Supplement: Supplementary file 4 — Correlations between kidney function parameters and T2DM and obesity related parameters at baseline in the total study population. [file 41387_2024_285_MOESM4_ESM.docx]

**Table S2.** Correlations between kidney function parameters and T2DM and obesity related parameters at baseline in the total study population.

|  | | **HbA1c** | **Fasting glucose** | **Fasting insulin** | **BMI** |
| --- | --- | --- | --- | --- | --- |
| **eGFR** | ρ Pearson | -0.143^*^ | -0.133^*^ | -0.060 | -0.033 |
|  | Sig. | <0.001 | <0.001 | 0.058 | 0.295 |
| **uACR** | ρ Pearson | 0.327^*^ | 0.308^*^ | 0.126^*^ | 0.137^*^ |
|  | Sig. | <0.001 | <0.001 | <0.001 | <0.001 |

Differences were considered to be significant when (*) p < 0.001.

T2DM; type 2 diabetes mellitus; eGFR, estimated glomerular filtration rate; uACR, urine albumin-creatinine ratio; BMI, body max index; HbA1c, glycated hemoglobin.
