# Supplementary material for: Mediterranean diet as a strategy for preserving kidney function in patients with coronary heart disease with type 2 diabetes and obesity: a secondary analysis of CORDIOPREV randomized controlled trial
Source: Nutr Diabetes. 2024 May 16;14:27. doi: 10.1038/s41387-024-00285-3 (PMC11099022; doi:10.1038/s41387-024-00285-3)
Supplement: Supplementary file 5 — Antidiabetic and antihypertensive treatment regimens at baseline and after 5-years of follow-up [file 41387_2024_285_MOESM5_ESM.docx]

|  | | **Non-Obesity/Non-T2DM** | | | | | | **Obesity/Non-T2DM** | | | | | |
| --- | --- | --- | --- | --- | --- | --- | --- | --- | --- | --- | --- | --- | --- |
|  |  | ***Low-Fat diet*** | | | ***Mediterranean diet*** | | | ***Low-Fat diet*** | | | ***Mediterranean diet*** | | |
|  |  | Baseline | After 5-y | P value | Baseline | After 5-y | P value | Baseline | After 5-y | p | Baseline | After 5-y | P value |
| **Anti-hypertensive drugs (%)** | ACE inhibitor or ARB | 81.6 | 75.9 | 0.499 | 73.5 | 74.3 | 0.961 | 81.3 | 82.3 | 0.867 | 80.4 | 80.4 | 1.000 |
|  | Calcium channel blockers | 21.8 | 21.8 | 0.999 | 15.4 | 21.4 | 0.357 | 21.9 | 27.1 | 0.474 | 26.2 | 26.2 | 0.999 |
|  | Beta blockers | 80.5 | 74.7 | 0.510 | 82.1 | 74.4 | 0.074 | 77.1 | 77.1 | 0.999 | 81.3 | 75.7 | 0.253 |
|  | Diuretics | 36.8 | 27.6 | 0.272 | 39.3 | 39.3 | 0.976 | 49.0 | 44.8 | 0.712 | 43.9 | 48.6 | 0.620 |
| **Anti-diabetic drugs (%)** | Metformin | 0.0 | 1.1 | 0.999 | 0.0 | 2.6 | 0.255 | 0.0 | 3.1 | 0.241 | 0.0 | 2.8 | 0.252 |
|  | Insulin | 0.0 | 0.0 | 1.000 | 0.0 | 0.0 | 1.000 | 0.0 | 0.0 | 1.000 | 0.0 | 0.0 | 1.000 |
|  | Metformin plus others | 0.0 | 0.0 | 1.000 | 0.0 | 0.0 | 1.000 | 0.0 | 0.0 | 1.000 | 0.0 | 0.0 | 1.000 |
|  | Insulin plus others | 0.0 | 0.0 | 1.000 | 0.0 | 0.0 | 1.000 | 0.0 | 0.0 | 1.000 | 0.0 | 0.9 | 0.999 |

**Table S3.** Antidiabetic and antihypertensive treatment regimens at baseline and after 5-years of follow-up

|  | | **Non-Obesity/T2DM** | | | | | | **Obesity/T2DM** | | | | | |
| --- | --- | --- | --- | --- | --- | --- | --- | --- | --- | --- | --- | --- | --- |
|  |  | ***Low-Fat diet*** | | | ***Mediterranean diet*** | | | ***Low-Fat diet*** | | | ***Mediterranean diet*** | | |
|  |  | Baseline | After 5-y | P value | Baseline | After 5-y | P value | Baseline | After 5-y | P value | Baseline | After 5-y | P value |
| **Anti-hypertensive drugs (%)** | [ACE inhibitor](about:blank) or ARB | 89.0 | 81.3 | 0.203 | 84.6 | 82.4 | 0.999 | 85.3 | 82.7 | 0.593 | 89.1 | 91.2 | 0.784 |
|  | Calcium channel blockers | 34.1 | 31.9 | 0.912 | 27.5 | 38.5 | 0.125 | 36.0 | 38.7 | 0.717 | 39.4 | 42.3 | 0.748 |
|  | Beta blockers | 76.9 | 69.2 | 0.345 | 80.2 | 71.4 | 0.328 | 84.7 | 80.7 | 0.394 | 78.8 | 81.0 | 0.847 |
|  | Diuretics | 59.3 | 52.7 | 0.495 | 57.1 | 54.9 | 1.000 | 66.7 | 68.7 | 0.796 | 62.0 | 69.3 | 0.270 |
| **Anti-diabetic drugs (%)** | Metformin | 23.1 | 18.7 | 0.610 | 30.8 | 22.0 | 0.275 | 24.0 | 24.7 | 1.000 | 19.7 | 15.3 | 0.408 |
|  | Insulin | 1.1 | 0.0 | 0.999 | 5.5 | 4.4 | 1.000 | 5.3 | 6.0 | 1.000 | 4.4 | 2.9 | 0.738 |
|  | Metformin plus others | 16.5 | 18.7 | 0.817 | 14.3 | 22.0 | 0.263 | 18.7 | 22.0 | 0.564 | 21.2 | 27.0 | 0.319 |
|  | Insulin plus others | 9.9 | 15.4 | 0.355 | 11.0 | 19.8 | 0.132 | 16.0 | 19.3 | 0.543 | 21.2 | 27.0 | 0.319 |

Data are percentage of participants. P value for comparisons between groups calculated with Chi-square tests. P < 0.05, baseline vs 5-years of follow-up.

T2DM, type 2 diabetes mellitus; ACE, angiotensin-converting enzyme; ARB, angiotensin-receptor blockers; Anti-diabetic drugs regimens are: Metformin means patients with metformin as unique anti-diabetic treatment; Insulin means patients with insulin as unique anti-diabetic treatment; Metformin plus others means patients with metformin plus other oral anti-diabetic treatment (sulfonylureas, thiazolidinediones, or DPP-4 inhibitors); Insulin plus others means patients with insulin plus other oral anti-diabetic treatment (sulfonylureas, thiazolidinediones, or DPP-4 inhibitors).
