# Supplementary material for: Mediterranean diet as a strategy for preserving kidney function in patients with coronary heart disease with type 2 diabetes and obesity: a secondary analysis of CORDIOPREV randomized controlled trial
Source: Nutr Diabetes. 2024 May 16;14:27. doi: 10.1038/s41387-024-00285-3 (PMC11099022; doi:10.1038/s41387-024-00285-3)
Supplement: Supplementary file 6 — Mean baseline values and changes in adherence after 5 years of dietary intervention. [file 41387_2024_285_MOESM6_ESM.docx]

**Table S4.** Mean baseline values and changes in adherence after 5 years of dietary intervention.

|  |  | **Non-Obesity/**  **Non-T2DM** | **Obesity/**  **Non-T2DM** | **Non-Obesity/**  **T2DM** | **Obesity/**  **T2DM** | ***p***  ***value*** |
| --- | --- | --- | --- | --- | --- | --- |
| **MEDAS** | **Baseline** | 9.10 ± 2.11 | 8.94 ± 1.91 | 8.93 ± 1.96 | 8.78 ± 1.90 | 0.650 |
|  | **Δchange** | 2.59 ± 2.43* | 2.43 ± 2.32* | 2.42 ± 2.22* | 2.46 ± 2.11* | 0.945 |
| **Low-fat diet screener** | **Baseline** | 3.98 ± 1.67 | 3.84 ± 1.72 | 3.92 ± 1.55 | 3.81 ± 1.52 | 0.877 |
|  | **Δchange** | 3.28 ± 1.90* | 3.11 ± 2.31* | 3.36 ± 2.11* | 3.04 ± 1.83* | 0.626 |

Data are presented as mean and Δchange produced between post- and preintervention ± standard error of the mean. Adherence to the Mediterranean diet is assessed by MEDAS (14-item Mediterranean Diet Adherence Screener) in the Mediterranean diet group (n =447) and adherence to the low-fat diet is assessed by a 9-item dietary screener with the low-fat diet guidelines (n=406). Differences were significant when p < 0.05. * Significant differences between post and pre-intervention.
